# Rapid Gene Concatenation for Genetic Rescue of Multigene Mutants in *Candida albicans*

**DOI:** 10.1128/mSphere.00169-18

**Published:** 2018-04-25

**Authors:** Manning Y. Huang, Carol A. Woolford, Aaron P. Mitchell

**Affiliations:** a Department of Biological Sciences, Carnegie Mellon University, Pittsburgh, Pennsylvania, USA; Duke University Medical Center

**Keywords:** CRISPR, *Candida albicans*, genetics

## Abstract

The biological function of a gene is often probed through its interactions with other genes. This general approach has been especially useful to build knowledge about poorly understood genes upon the bedrock of well-characterized genes. Genetic interaction analysis requires the construction of strains with mutations in two or more genes. Single-gene mutants of microbial pathogens are generally validated through introduction of a wild-type copy of the affected gene to create a complemented or reconstituted strain, followed by testing for restoration of a wild-type phenotype. This practice, formalized as one of Falkow’s “molecular Koch’s postulates” ensures that the phenotype of the mutant depends upon the known mutation. However, multigene mutants are seldom validated because of the labor required to assemble multiple genomic segments into a vector that can be introduced into the mutant strain. We present here an approach, concatemer assembly for rescue of mutant abilities (CARMA), that circumvents this impediment through an *in vivo* recombinational assembly strategy that does not require cloning at all. Our results show that CARMA allows genetic rescue of two double-gene mutant strains of the fungal pathogen Candida albicans.

**IMPORTANCE** Our understanding of new genes is often built upon the knowledge of well-characterized genes. One avenue toward revealing such connections involves creation of strains with mutations in two or more defined genes to permit genetic interaction analysis. Strain manipulations can yield unexpected mutations at loci outside the defined targeted genes. In this report, we describe a method for rapid validation of multigene mutants, thus allowing an appraisal of the contribution of the defined targeted genes to the strain’s phenotype.

## Abstract (precis)

Our understanding of new genes is often built upon the knowledge of well-characterized genes. One avenue toward revealing such connections involves creation of strains with mutations in two or more defined genes to permit genetic interaction analysis. Strain manipulations can yield unexpected mutations at loci outside the defined targeted genes. In this report, we describe a method for rapid validation of multigene mutants, thus allowing an appraisal of the contribution of the defined targeted genes to the strain’s phenotype.

## INTRODUCTION

For many organisms, our understanding of gene function is based upon analysis of engineered mutations at predetermined genomic sites. The manipulations that yield engineered mutations can also yield adventitious mutations elsewhere in the genome. For this reason, when characterizing a defined mutant strain, there is a concern that its phenotype may be due to adventitious mutations, rather than the engineered mutation. For microbial pathogens, mutant strain characterization can be extensive, involving, for example, animal infection models. Moreover, strain phenotypes may appear to be complex because many mechanistic underpinnings of virulence have yet to be discovered. For these organisms, the possible contributions of adventitious mutations to the phenotype are often assessed through analysis of a complemented or reconstituted strain, in which a wild-type copy of the gene that had been altered by engineering is introduced at a distant genomic site (complementation) or at the native locus (reconstitution). The importance of including complemented or reconstituted strains in pathogen phenotypic characterization was emphasized as the third postulate among the cornerstone “molecular Koch’s postulates” coined by Falkow (1).

Once there is a panel of well-characterized genes in an organism, it is useful to build upon that knowledge in the discovery or characterization of new genes. The approach of genetic interaction analysis, also called epistasis analysis, is a widely used strategy that can connect the functions of two genes. In premier genetic organisms like Saccharomyces cerevisiae, systematic gene interaction analysis has been applied on a large scale to reveal new gene functions and novel connections among seemingly distinct biological processes (2). Our organism of interest, Candida albicans, is not as amenable to genetic manipulation as S. cerevisiae, but nonetheless, it has a growing panel of characterized genes that provide the foundation for genetic interaction analysis. In fact, the use of double and multigene mutants in C. albicans to draw functional inferences has a long history (3–11). Studies of C. albicans double and multigene mutants have provided key insights into functional redundancy, pathway relationships, and major effectors that mediate pathway outputs. The analysis of double and multigene mutants has been of exceptional value for the understanding of C. albicans, as it has for diverse other organisms.

It is likely that analysis of C. albicans multigene mutants will have an increasingly prominent role in elucidation of the basis for key traits such as pathogenicity and drug resistance. This expectation is based upon the value of the information from these strains as well as the increasing ease with which multigene mutants can be created. Strain construction in C. albicans has been accelerated through implementation of clustered regularly interspaced short palindromic repeat (CRISPR)-Cas9-based methods (12), and recently, a gene drive system designed specifically for creation of double mutant strains (10). Unfortunately, the development of multigene complementation or reconstitution strategies, which we refer to together as genetic rescue strategies, has not kept pace with these developments. Strain validation through genetic rescue generally requires vector-based cloning, an often tedious procedure when multiple genes must be introduced together.

Here, we present a genetic rescue strategy that relies on recombinational assembly in C. albicans of PCR products. Integration of the concatenated assembly into a mutant strain is augmented by CRISPR-Cas9 cleavage at the targeted locus. Then, the mutant strain that carries the concatenated assembly is tested to see whether a wild-type phenotype is restored. We report proof-of-principle studies for phenotypic rescue of auxotrophic markers and for mutants defective for biofilm formation and filamentation. This approach, which we call concatemer assembly for rescue of mutant abilities (CARMA), has the potential to accelerate multigene mutant validation in C. albicans and other organisms.

## RESULTS

**FIG 1:**
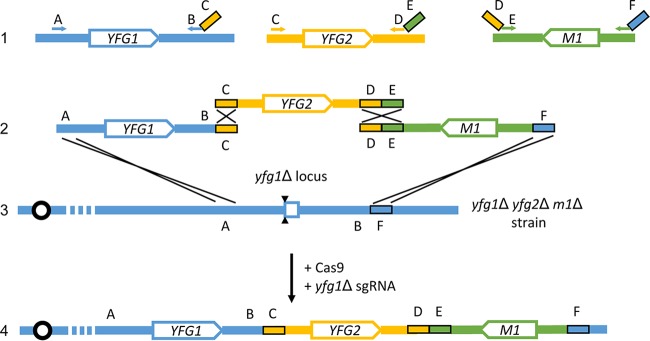
Strategy for concatemer assembly for rescue of mutant abilities (CARMA). Given a double mutant strain with the genotype *yfg1*Δ/Δ *yfg2*Δ/Δ with an available selectable marker *M1*, both *YFG1* and *YFG2* may conceptually be reintroduced at either the *yfg1*Δ (depicted in line 2 and line 3) or *yfg2*Δ locus. Cassettes containing *YFG1* and *YFG2* are generated by PCR from wild-type genomic DNA (line 1). The *YFG1* cassette with sequences shown in blue is amplified with the primers indicated at the A and BC regions. It contains a segment of the *YFG1* promoter, the *YFG1* open reading frame (ORF), and terminator. Sequence C, containing homology to the *YFG2* cassette, is introduced into the *YFG1* cassette using the long primer indicated at the BC region. The *YFG2* cassette with sequences shown in gold is amplified with the primers indicated at the C and DE regions. It contains a segment of the *YFG2* promoter, the *YFG2* ORF, and terminator. Sequence E, containing homology to the *M1* marker cassette, is introduced into the *YFG2* cassette using the long primer indicated at the DE region. The *M1* marker cassette with sequences shown in green is amplified from a plasmid containing the *M1* marker with the primers indicated at the DE and F regions. Sequence D, containing homology to the terminator region of *YFG2*, is introduced into the *M1* cassette using the long primer indicated at the DE region. Sequence F, containing homology to a region downstream of *YFG1* is introduced into the *M1* cassette using the long primer indicated at the F region. All three cassettes are transformed into the *yfg1*Δ/Δ *yfg2*Δ/Δ *m1*Δ/Δ strain under M1 selection, along with DNA cassettes expressing *CAS9* and a single guide RNA targeting the *yfg1*Δ locus. The double-strand break introduced by Cas9 complexed with the *yfg1*Δ targeting sgRNA is indicated by two black triangles. Expected homologous recombination events are depicted as single crosses, and together, they should yield a concatenated locus containing *YFG1*, *YFG2*, and *M1* (line 4). Boxes C, D, E, and F each indicate segments of 80 bp. Where possible, homology between PCR cassettes is maximized. Sequences D and E together provide 160 bp of homology between the *YFG2* and *M1* cassettes. (In pilot experiments, we could not generate integrated concatemers with only 80 bp of homology between the *YFG2* and *M1* cassettes.) Sequences C and F provide 80 bp of homology to the *YFG1* cassette and *yfg1*Δ locus, respectively. For construction of the *ARG4*:*LEU2*:*HIS1* allele, the A-*YFG1* interval provided 419 bp of homology. For construction of the *UME6*:*BRG1*:*HIS1* allele, the A-*YFG1* interval provided 403 bp of homology. In our experiments, we avoided extending the homology between the *YFG1* and *YFG2* cassettes to 160 bp in length by adding 80 bp of homology at sequence B. We were concerned that the presence of sequence B in the *YFG2* cassette could allow integration of only *YFG2* and *M1* downstream of the *yfg1*Δ deletion scar in the B-F interval.

We developed a multigene rescue strategy that employs three DNA segments (Fig. 1). In this illustration, we seek to introduce functional *YFG1* and *YFG2* genes to assay phenotypic rescue of a *yfg1*Δ/Δ *yfg2*Δ/Δ double mutant. We include the marker *M1* in order to select for transformation and integration of a concatemer of the three segments. Each segment is synthesized through a PCR templated by the genome or an existing plasmid, thus eliminating any need for custom cloning. The segments have homology at their ends to one another or to the genome (regions A to F in Fig. 1), so that cellular homologous recombination machinery can assemble the segments in a predictable order. We refer to an organization of homology that directs concatenation of the DNA segments as “concatenating homology.”

The first (leftmost) segment includes one of the coding regions, *YFG1*, that corresponds to one of the deletions in the *yfg1*Δ/Δ *yfg2*Δ/Δ double mutant (Fig. 1). It also includes *YFG1* 5′ and 3′ flanking regions. The overall assembly will be directed to integrate at the *YFG1* genomic locus to replace a *yfg1*Δ coding region deletion allele. Therefore, the *YFG1* 5′ flanking region does not need to include all sequences necessary for proper expression; integration by recombination at the A region will join the *YFG1* DNA to the complete genomic 5′ flanking region that extends to the left in the diagram. The segment needs to include sufficient 3′ flanking sequences for *YFG1* expression, extending to the B region. This requirement arises because the 3′ flanking end of the *YFG1* segment will be fused to novel sequences from the *YFG2* locus, the C region, in the integrated assembly.

The second (middle) segment includes the second coding region, *YFG2*, that corresponds to a deletion in the *yfg1*Δ/Δ *yfg2*Δ/Δ double mutant (Fig. 1). This segment includes *YFG2* 5′ and 3′ flanking regions that extend to regions labeled C and D. These flanking regions must include all sequences necessary for proper expression because both regions are joined to novel neighboring sequences, the B and E regions, specified by the PCR primers.

The third (rightmost) segment includes a marker for selection, *M1*, along with all necessary 5′ and 3′ flanking sequences for expression (Fig. 1). It has homology to the *YFG2* segment (D-E region) at one end and to the genome neighboring the *yfg1*Δ allele (F region) at the other end. The 5′-to-3′ orientation of each gene (*M1*, *YFG1*, and *YFG2*) is arbitrary; the diagram illustrates the actual relative orientations of the genes in the experiments we present below.

Homologous recombination should not allow integration of *M1* on its own. However, the *YFG1* and *YFG2* segments may serve as adaptors to enable homologous recombination events to insert a *YFG1*:*YFG2*:*M1* concatenated assembly in place of the *yfg1*Δ allele (Fig. 1). Genomic integration of the concatemer may be stimulated by double-strand cleavage of the *yfg1*Δ allele directed by the CRISPR-Cas9 system (Fig. 1).

### Genetic rescue of *arg4*, *leu2*, and *his1* mutations in strain SN152.

We chose the popular laboratory strain SN152, which has *arg4*Δ/Δ, *leu2*Δ/Δ, and *his1*Δ/Δ mutations, for an initial test of the multigene rescue strategy. The strategy is expected to yield Arg^+^ Leu^+^ His^+^ transformants through recombinational concatemerization and integration of *ARG4*, *LEU2*, and *HIS1* cassettes. We designed three cassettes with concatenating homology as depicted in Fig. 1, using the C. albicans *ARG4* gene in the position of *YFG1*, the Candida albicans *LEU2* gene in the position of *YFG2*, and the Candida dubliniensis *HIS1* gene in the position of *M1*. Integration of the cassettes was targeted by homology to the *arg4*Δ::*dpl200* mutant locus in the SN152 genome, and transformants were selected through acquisition of only a His^+^ phenotype. If the other two genes were assembled into an integrating concatemer, then His^+^ transformants should be Arg^+^ and Leu^+^ as well. Transformation of approximately 3 µg of each cassette yielded seven His^+^ transformants from three separate transformations (Table 1). One transformant could not be propagated on media selective for His^+^ strains and was not studied further. Five of the remaining six transformants grew when replica plated to minimal SD medium and thus were Arg^+^ Leu^+^ His^+^. This observation indicates that the *ARG4*, *LEU2*, and *HIS1* genes were all maintained. Thus, genetic rescue with three cassettes is possible, though the transformant recovery frequency is low (13, 14).

**TABLE 1:** SN152 His^+^ Leu^+^ Arg^+^ transformation outcomes

Expt or parameter	No. of His^+^ colonies with no sgRNA	No. of His^+^ colonies with sgRNA
1	4	831
2	3	716
3	0	402

Avg ± SD	2.33 ± 2.08	650 ± 222

% prototrophic	83	94 ± 2.7

The CRISPR-Cas9 system has been used to increase the rate of DNA integration through generation of a genomic double-strand break that stimulates homology-directed repair. To test the effect of CRISPR-Cas9 on concatemer integration, we included DNA cassettes specifying Cas9 and a single guide RNA (sgRNA) that targets the *arg4*Δ::*dpl200* locus in our transformation mixture. These components increased the recovery of His^+^ transformants by roughly 300-fold (Table 1). Approximately 94% of His^+^ transformants grew when replica plated to SD medium, thus indicating once again that the *ARG4*, *LEU2*, and *HIS1* genes were all maintained. This result indicates that multiple cassettes with concatenating homology may successfully integrate with selection for only a single cassette and that integration is greatly enhanced by the introduction of a genomic double-strand break at the integration site.

### Genetic rescue of a *ume6*Δ/Δ *brg1*Δ/Δ mutant strain.

**FIG 2:**
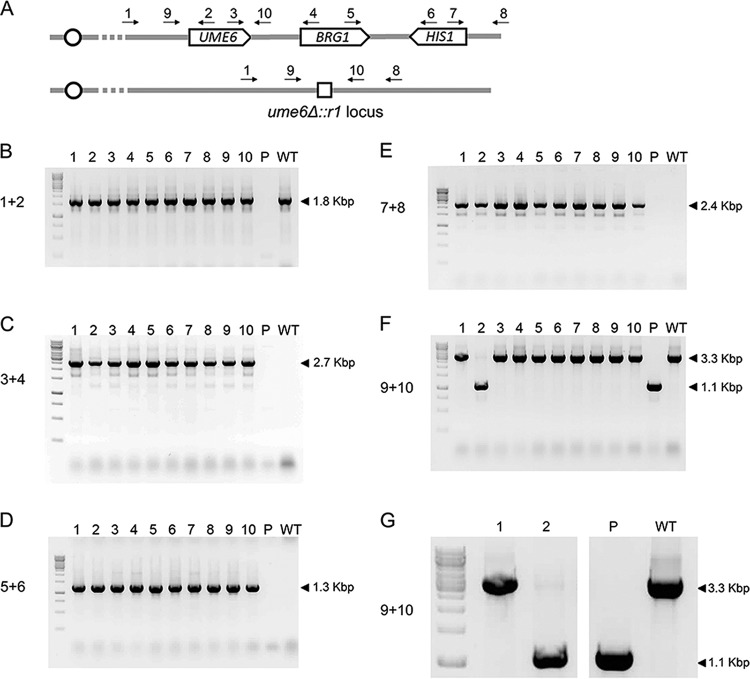
Genotyping *UME6*:*BRG1*:*HIS1* candidates. (A) Primer pairs for detection of *UME6*, *BRG1*, and *HIS1*. The *UME6*:*BRG1* locus is depicted in the first line with the expected location and integration of *UME6*, *BRG1*, and *HIS1* depicted. Primers 1 and 2 are used for detection of *UME6* at the *ume6*Δ::*r1* locus. Primer 1 anneals upstream of any predicted recombinational junction. Primers 3 and 4 are used to detect *BRG1* integration downstream of *UME6*. Primers 5 and 6 are used to detect *HIS1* integration downstream of *BRG1*. Primers 7 and 8 are used to detect *HIS1* at the *ume6*Δ::*r1* locus. Primer 8 sits downstream of any predicted recombinational junction. The *ume6*Δ::*r1* locus is depicted on the second line, with the genetic scar consisting of a single repeat from the *r1HIS1r1* cassette indicated by a small white box. Primers 9 and 10 bind upstream and downstream of the *ume6*Δ::*r1* locus and can be used for detection of the *ume6*Δ::*r1* allele. (B) PCR products for detection of *UME6* at its native locus using primer pair 1 plus 2. Amplification of a 1.8-kbp band is consistent with the presence of *UME6* at its native locus. (C) PCR products for detection of the *UME6*:*BRG1* junction using primer pair 3 plus 4. Amplification of a 2.7-kbp band is consistent with the presence of *BRG1* downstream of *UME6*. (D) PCR products for detection of the *BRG1*:*HIS1* junction using primer pair 5 plus 6. Amplification of a 1.3-kbp band is consistent with the presence of *HIS1* downstream of *BRG1*. (E) PCR products for detection of integration of *HIS1* at the *ume6*Δ::*r1* locus using primer pair 7 plus 8. Amplification of a 2.4-kbp band is consistent with the presence of *HIS1* at the *ume6*Δ::*r1* locus. (F) PCR products for detection of homozygous integration using primer pair 9 plus 10. Amplification of a 1.1-kbp band suggests the presence of a *ume6*Δ::*r1* allele (see the bottom line in panel A). Amplification of a 3.3-kbp band is consistent with the presence of either a wild-type (WT) *UME6* or concatenated *UME6*:*BRG1* allele (see the top line in panel A). A 1.1-kbp band is observed in the parental strain and in the strain in lane 2, indicating the presence of at least one copy of the *ume6*Δ::*r1* allele. The absence of a 1.1-kbp band in the strains in lanes 1 and 3 to 10 suggests homozygous integration of the concatenated allele. (G) Expanded insert of panel F for detection of homozygous integration. Lane 2 contains a 3.3-kbp band which is absent in the parental strain. The 3.3-kbp band in lane 2 is consistent with integration of at least one *UME6* allele, which suggests that this strain underwent heterozygous integration of the *UME6*:*BRG1* concatemer.

We also tested our genetic rescue strategy with a *ume6*Δ/Δ *brg1*Δ/Δ double mutant strain. *UME6* and *BRG1* encode transcription factors that promote C. albicans filamentation and biofilm formation (15–18). These two genes have large promoter and regulatory regions, and cloning of the two large regions for a conventional complementing construct could be laborious. We designed three PCR products with concatenating homology (Fig. 1): a *UME6* segment (*YFG1*), a *BRG1* segment (*YFG2*), and a C. dubliniensis *HIS1* segment (*M1*). Integration of the concatenated cassettes was targeted to the *ume6*Δ::*r1* locus. The *ume6*Δ::*r1* locus has a deletion of the *UME6* coding region; it contains the *UME6* upstream and downstream regions flanking a 360-bp segment (designated *r1*) of vector pRS424 (19). The strain was transformed with approximately 3 µg of each PCR product along with cassettes specifying Cas9 and a single guide RNA that targets the *ume6*Δ::*r1* allele. We recovered more than 200 His^+^ transformants from a single transformation. Ten transformants were selected for PCR genotyping. All 10 transformants yielded the PCR product (primers 1 plus 2 [Fig. 2A and B]) expected from joining of the *UME6* coding region with the *UME6* 5′ flanking region. All 10 transformants also yielded the PCR product (primers 3 plus 4 [Fig. 2A and C]) expected from the presence of the *UME6* coding region adjacent to the *BRG1* coding region. In addition, all 10 transformants yielded the PCR product (primers 5 plus 6 [Fig. 2A and D]) expected from the presence of the *BRG1* coding region adjacent to the *HIS1* coding region. Finally, all 10 transformants yielded the PCR product (primers 7 plus 8 [Fig. 2A and E]) expected from the presence of the *HIS1* coding region adjacent to the *UME6* 3′ region. These results are consistent with the structure expected for integration of a concatenated *UME6*:*BRG1*:*HIS1* DNA segment at the *ume6*Δ::*r1* locus in the genome. Therefore, these genotyping results indicate that concatemer integration occurred in the majority of transformants.

We anticipated that we may recover homozygous integrants as a result of Cas9-sgRNA cleavage of both *ume6*Δ::*r1* alleles (12). PCR analysis indicated that 9 of the 10 genotyped transformants lacked a detected *ume6*Δ::*r1* allele and thus were likely homozygous integrants (primers 9 plus 10 [Fig. 2A and F]). One genotyped transformant was apparently heterozygous (primers 9 plus 10 [Fig. 2A, F, and G]). These results argue that the majority of our transformants were homozygous for the recombinational concatemer, integrated to replace the *ume6*Δ::*r1* locus in the genome.

For phenotypic rescue assays, we focused on one transformant, strain MH281, that carried the integrated *UME6*:*BRG1*:*HIS1* concatenation product and was presumably homozygous due to the absence of a *ume6*Δ::*r1* allele. We refer to this strain as the validation strain. Three assays were used to assess phenotypic rescue: filamentation, biofilm formation, and expression of *UME6*- and *BRG1-*responsive genes (11, 15–18). We compared these phenotypes in the wild-type strain, a *ume6*Δ/Δ single mutant, a *brg1*Δ/Δ single mutant, a *ume6*Δ/Δ *brg1*Δ/Δ double mutant, and the *UME6*:*BRG1* validation strain. We sought to use strains with matched nutritional requirements; therefore, for example, the *ume6*Δ/Δ *brg1*Δ/Δ strain was a His^+^ derivative of the His^−^ parental *ume6*Δ/Δ *brg1*Δ/Δ double mutant that had been transformed to create the validation strain. Similarly, the other strains had auxotrophies complemented.

**FIG 3:**
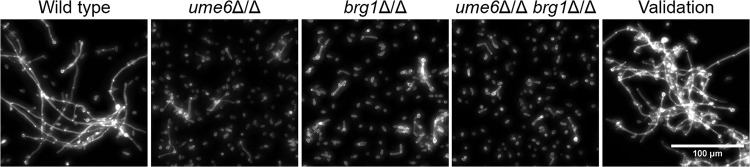
Filamentation phenotypes of mutant and validation strains. Fluorescence images of cells with Calcofluor white staining after 4 h of growth in YPD plus serum. The wild-type (WT) SN250, *ume6*Δ/Δ mutant, *brg1*Δ/Δ mutant, *ume6*Δ/Δ *brg1*Δ/Δ double mutant, and *UME6*:*BRG1* validation strains were assayed for phenotypic rescue of filamentation under planktonic growth conditions. The wild-type strain forms long filamentous hyphae, whereas the *ume6*Δ/Δ mutant, *brg1*Δ/Δ mutant, and *ume6*Δ/Δ *brg1*Δ/Δ double mutant grow primarily as yeast-form cells. The *UME6*:*BRG1* validation strain forms long filamentous hyphae comparable to those of WT cells.

Filamentation was assayed by microscopic observation after growth in yeast extract-peptone-dextrose (YPD) plus serum at 37°C (Fig. 3). The wild-type strain formed long hyphae with few detached yeast-form cells. The *ume6*Δ/Δ *brg1*Δ/Δ double mutant failed to form filaments under these conditions. The *ume6*Δ/Δ and *brg1*Δ/Δ mutants each formed short hyphae intermixed with a large proportion of yeast-form cells. The *UME6*:*BRG1* validation strain, like the wild type, formed long hyphae and few yeast-form cells. These observations indicate that the inability of the double mutant strain to undergo filamentation is due to the *ume6*Δ/Δ *brg1*Δ/Δ genotype, because (i) each single mutant has a filamentation defect and (ii) the *UME6*:*BRG1* concatemer restores filamentation when introduced into the *ume6*Δ/Δ *brg1*Δ/Δ background.

**FIG 4:**
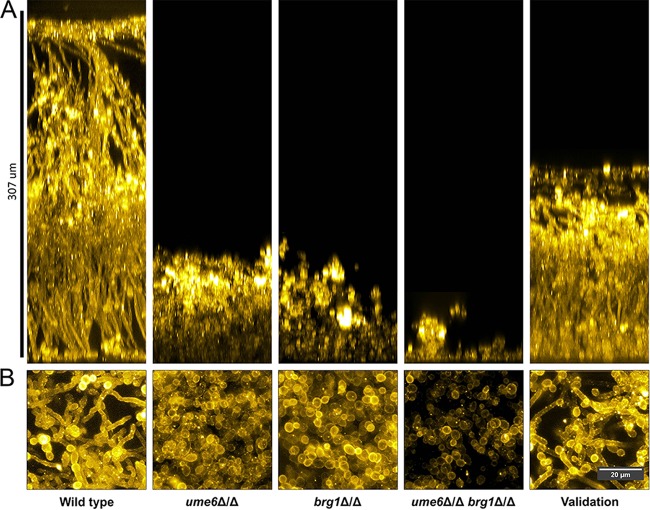
Side-view and apical-view projections of mutant and validation strain biofilms. The WT SN250 strain, *ume6*Δ/Δ mutant, *brg1*Δ/Δ mutant, *ume6*Δ/Δ *brg1*Δ/Δ double mutant, and *UME6*:*BRG1* validation strains were assayed for phenotypic rescue of biofilm formation. (A) Lateral projections of C. albicans WT, mutant, and validation strain biofilms. A wild-type strain forms a large robust biofilm, while the *ume6*Δ/Δ, *brg1*Δ/Δ, and *ume6*Δ/Δ *brg1*Δ/Δ mutant strains form defective biofilms. The *UME6*:*BRG1* validation strain forms a biofilm with more than half the thickness of the WT biofilm. (B) Apical-view projections of representative sections of each biofilm. A wild-type strain forms a robustly filamentous biofilm, whereas *ume6*Δ/Δ, *brg1*Δ/Δ, and *ume6*Δ/Δ *brg1*Δ/Δ mutant strains form biofilms with largely reduced filamentation. The *UME6*:*BRG1* strain forms a biofilm with visibly filamentous cells.

Biofilm formation was assayed by confocal microscopy after growth on silicone squares in YPD alone at 37°C for 24 h (Fig. 4). The wild-type strain formed a robust biofilm of 300 µm in depth with abundant hyphae evident in both side-view and apical-view projections. The *ume6*Δ/Δ *brg1*Δ/Δ mutant was severely defective in biofilm formation: few cells adhered to the silicone surface, and no hyphae were apparent (Fig. 4A and B). The *ume6*Δ/Δ and *brg1*Δ/Δ single mutants were also defective, yielding biofilms composed exclusively of yeast-form cells and with a depth of ~50 to 70 µm, as reported previously for 48-h biofilms (11). The *UME6*:*BRG1* validation strain produced a biofilm with an intermediate depth, a depth between that of the wild-type strain and those of the single mutant strains. Abundant hyphae were evident in side-view and apical-view projections (Fig. 4A and B). These observations indicate that the inability of the double mutant strain to produce a biofilm is due to the *ume6*Δ/Δ *brg1*Δ/Δ genotype, because (i) each single mutant has a biofilm defect, and (ii) the *UME6*:*BRG1* concatemer restores biofilm when introduced into the *ume6*Δ/Δ *brg1*Δ/Δ background. However, the *UME6*:*BRG1* concatemer enables only partial rescue of the biofilm defect, because the validation strain does not produce as extensive a biofilm as the wild-type strain does.

**FIG 5:**
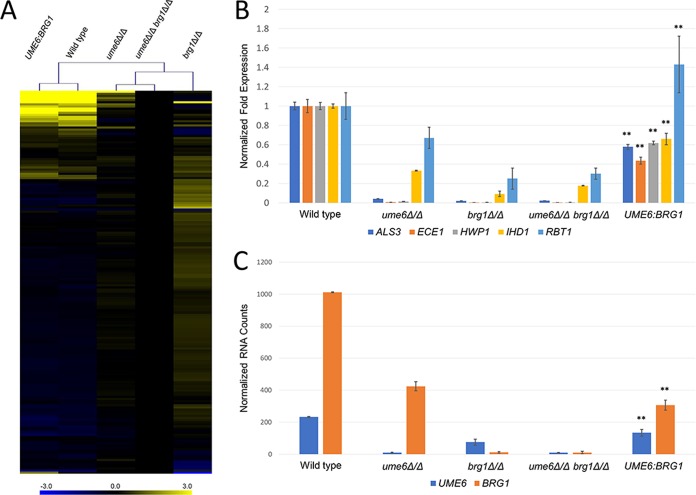
RNA expression levels in mutant and validation strains. RNA was extracted from WT SN250, *ume6*Δ/Δ mutant, *ume6*Δ/Δ *brg1*Δ/Δ double mutant, and *UME6*:*BRG1* validation strains grown for 4 h in YPD at 37°C and then analyzed with a NanoString nCounter. Three biological replicates were assayed, and the average of all replicates was used for further analysis. *brg1*Δ/Δ mutant gene expression was drawn from Woolford et al., assayed under the same conditions as this experiment (4 h YPD, 37°C) and analyzed using the same NanoString code set (11). (A) A heat map shows log_2_ fold expression of environmentally responsive genes between strains normalized against expression in a *ume6*Δ/Δ *brg1*Δ/Δ double mutant. Log_2_ fold expression is depicted on a color gradient ranging between −3.0 (blue) and +3.0 (yellow). The tree depicts clustering of strains according to an algorithm for minimum Manhattan distance (MeV software). The *ume6*Δ/Δ mutant, *brg1*Δ/Δ mutant, and *ume6*Δ/Δ *brg1*Δ/Δ double mutant cluster, showing similar profiles with reduced expression of environmentally responsive genes. The *UME6*:*BRG1* validation strain clusters with the wild-type strain, showing similar expression profiles. (B) Mean fold change in the expression of hypha-associated genes *ALS3*, *ECE1*, *HWP1*, *IHD1*, and *RBT1* in mutant strains and validation strain normalized against expression in the wild-type strain. Values are means ± standard deviations (SDs) (error bars). Mean fold change values that are significantly different (*P* < 0.01 by Tukey-Kramer test) from the values for the mutant strains are indicated by two asterisks. (C) Mean normalized counts of *UME6* and *BRG1* mRNA between WT, mutant, and validation strains. Values are means ± SDs. Mean RNA counts that are significantly different (*P* < 0.01 by Tukey-Kramer test) from the mean RNA counts of all other strains are indicated by two asterisks.

Gene expression was measured by Nanostring on RNA samples from cells grown for 4 h in YPD at 37°C. The probe set assayed RNA levels for a panel of 182 genes, including 166 environmentally responsive genes (11). The wild-type strain had significantly different RNA levels (>2-fold change; *P* < 0.05) than the *ume6*Δ/Δ *brg1*Δ/Δ double mutant for 18 genes in addition to the deleted genes *UME6* and *BRG1* (Fig. 5A). Almost all of these RNA levels were also significantly different in the wild-type strain compared to the *ume6*Δ/Δ and *brg1*Δ/Δ single mutant strains. The affected genes included the core hyphal-associated genes *ALS3*, *ECE1*, *HWP1*, *IHD1*, and *RBT1* (Fig. 5B), as expected (15–18). The validation strain expressed the affected RNAs at levels similar to those of the wild-type strain (Fig. 5A). Most of the core hyphal-associated genes had RNA levels of roughly 50% of the level in the wild-type strain. These observations confirm that the concatenated *UME6*:*BRG1* construct partially rescues the biofilm defect of the *ume6*Δ/Δ *brg1*Δ/Δ double mutant.

Partial rescue by *UME6*:*BRG1* may result from incompletely restored expression of the *UME6* or *BRG1* gene. We were concerned in particular about *BRG1* expression for two reasons. First, its genomic 5′ flanking region extends for over 10 kbp to the next open reading frame, but only 1.6 kbp had been included in our concatenation cassette. Second, Brg1 is a positive regulator of *UME6* expression, so a reduction in *BRG1* expression may cause a reduction in *UME6* expression as well (18). Our Nanostring measurements indicated that the validation strain expressed *BRG1* at ~30% of the wild-type level and expressed *UME6* at ~60% of the wild-type level (Fig. 5C). These results are consistent with the model that the *UME6*:*BRG1* concatenated construct allows partial phenotypic rescue because expression of the *BRG1* and *UME6* genes is only partially restored.

## DISCUSSION

Genetic interaction analysis is a vital tool to help decipher functional relationships in pathways, networks, and protein complexes (2). Central to this analysis is the ability to construct and validate multigene mutants. In this report, we have described a simple approach to introduce multiple DNA segments as an integrated genomic concatemer to generate a validation strain. The validation strain is then used in assays for rescue of multigene mutant phenotypes. We refer to the approach as concatemer assembly for rescue of mutant abilities (CARMA) in order to convey features of both the technical procedure and experimental objective.

The CARMA approach builds upon well-founded principles. Homologous recombination can seal a break in a target DNA, as demonstrated by Ma et al. with plasmid DNA in S. cerevisiae (20). Multiple DNA fragments with overlapping homology can be assembled through *in vivo* recombination in S. cerevisiae, as used for example in high-throughput construction of gene deletion cassettes (21) and in construction of a synthetic bacterial genome (22). A genomic CRISPR cut in a C. albicans mutant allele can enable integration of the wild-type allele, as shown by Nguyen et al. (23). We view CARMA as an amalgam of the principles demonstrated in these prior studies.

CARMA requires some attention to the extent of functional regulatory regions. Integration of the multigene concatemer at one of the mutant loci reconstitutes that gene with its native genomic 5′ flanking region. We chose to integrate specifically at the *ume6*Δ::*r1* locus because *UME6* is known to have a large 5′ regulatory region that includes 7 kbp or more upstream of the open reading frame (24, 25). Long 5′ regulatory regions are also found among other genes connected to filamentation, including *NRG1*, *HWP1*, and *ALS3* (24, 26, 27). The *BRG1* 5′ regulatory region has not been subjected to detailed analysis, and we included 1.6 kbp of 5′ flanking sequence in our genetic rescue segment. For many genes, we have found that ~1.6 kbp is sufficient for full rescue. However, we note that the *BRG1* 5′ region extends for more than 10 kbp before the next open reading frame, so it seems possible that it is yet another filamentation-related gene with a long 5′ regulatory region. We infer that 1.6 kbp of 5′ flanking sequence is not sufficient for full *BRG1* expression, because *BRG1* RNA levels were lower in the validation strain than in the wild-type strain. The reduced *BRG1* expression was nonetheless sufficient for substantial rescue of the double mutant phenotypes. Perhaps the best quantitative measures of function were the levels of expression of *UME6*- and *BRG1-*dependent target genes, which were significantly greater than those observed for either single mutant or the double mutant.

Note that, in all experiments reported here, the target integration loci are deletion alleles with fairly small relics of the initial marker; they are *yfg1*Δ::*dpl200* or *yfg1*Δ::*r1* alleles. Our preliminary studies suggest that a CARMA-like approach is less efficient with insertion-deletion alleles of structure *yfg1*Δ::*ARG4*. In addition, it is noteworthy that many mutant strains currently in use have different markers at the two alleles (genotype *yfg1*Δ::*ARG4/yfg1*Δ::*URA3*, for example). The recipient strains in the present study were all homozygous for identical mutant alleles at the target integration locus. Therefore, for existing *yfg1*Δ::*ARG4/yfg1*Δ::*URA3* strains, it may be necessary to use two sgRNA genes in order to direct integration to both alleles.

A few features of CARMA seem especially useful. First, all of the DNA molecules in the transformation reaction are PCR products, including the DNA segments destined to be assembled into the integrated concatemer, and the genes specifying Cas9 and the sgRNA, which are the original constructs used by Vyas et al. (12) used with our transient CRISPR protocol (13). This feature saves some time because the investigator circumvents cloning and screening for appropriate plasmids. This feature also overcomes the potential problem that a DNA fragment may be unstable in the intermediate host used for cloning. A second useful feature is that homozygous integrants can be recovered. This feature can eliminate partial phenotypic rescue due to gene dosage effects. Third, while in CARMA, each DNA segment corresponded to a complete coding region and flanking regions, concatenating homology of PCR products could also be used for construction of fusion genes, localized mutagenesis, mapping of functional promoter elements, or introduction of allelic variants.

## MATERIALS AND METHODS

### Strains, media, and transformations.

C. albicans strains were all archived in 15% glycerol stocks stored at −80°C. Strains were grown on solid yeast extract-peptone-dextrose (YPD) medium for 2 days at 30°C and then in liquid YPD medium overnight at 30°C with shaking prior to all assays and transformations. Transformations were performed using the lithium acetate transformation method supplemented with transient CRISPR-Cas9 system components (13). Transformants were selected on synthetic medium lacking specified auxotrophic supplements (2% dextrose, 0.67% Difco yeast nitrogen base without amino acids, and complete supplement mixture lacking the indicated amino acids). Replica plating and patching were performed using synthetic dextrose (SD) medium (2% dextrose, 0.67% Difco yeast nitrogen base without amino acids). All strains are listed in Table S1 in the supplemental material.

10.1128/mSphere.00169-18.1 TABLE S1  Candida albicans strains used in this study. Download TABLE S1, XLSX file, 0.01 MB. Copyright © 2018 Huang et al. 2018 Huang et al. This content is distributed under the terms of the Creative Commons Attribution 4.0 International license.

### Preparation of DNA cassettes and genotyping.

In preparation for the amplification of wild-type alleles, genomic DNA was prepared from the SC5314 strain by a modified version of the Hoffman and Winston procedure (28).

PCR was performed for 30 cycles for all PCRs according to the manufacturer’s protocols using TaKaRa *Ex Taq* DNA polymerase. All primer sequences are listed in Table S2.

10.1128/mSphere.00169-18.2 TABLE S2  Oligonucleotide primer sequences. Download TABLE S2, XLSX file, 0.01 MB. Copyright © 2018 Huang et al. 2018 Huang et al. This content is distributed under the terms of the Creative Commons Attribution 4.0 International license.

The C. albicans *ARG4* gene was amplified from SC5314 genomic DNA using primers CaARG4 MGC/F and CaARG4 3′->CaLEU2 5′/R. The C. albicans *LEU2* gene was amplified from SC5314 genomic DNA using primers CaLEU2 MGC/F and CaLEU2 3′->CdHIS1 for/R. The C. dubliniensis *HIS1* cassette was amplified from plasmid pSN52 (29) using primers CdHIS1 for->CaLEU2 3′/F and CdHIS1 rev->CaARG4 3′/R (29).

The CaARG4 3′->CaLEU2 5′/R primer contains 80 bp of homology to the upstream region of the C. albicans *LEU2* gene. The CaLEU2 3′->CdHIS1 for/R primer contains 80 bp of homology to the upstream region of the C. dubliniensis *HIS1* cassette. The CdHIS1 for->CaLEU2 3′/F primer contains 80 bp of homology to the downstream region of the C. albicans *LEU2* gene. The CdHIS1 rev->CaARG4 3′/R primer contains 80 bp of homology to the downstream region of the *ARG4* locus.

The C. albicans *UME6* gene was PCR amplified from SC5314 genomic DNA using primers UME6 MGC/F and UME6 3′->BRG1 5′/R. The C. albicans *BRG1* gene was PCR amplified from SC5314 genomic DNA using primers BRG1 MGC/F and BRG1 3′->CdHIS1 for/R. The C. dubliniensis *HIS1* cassette was amplified from plasmid pSN52 (29) using primers CdHIS1 for->BRG1 3′/F and CdHIS1 rev->UME6 3′/R (29).

The UME6 3′->BRG1 5′/R primer contains 80 bp of homology to the upstream region of the C. albicans *BRG1* gene. The BRG1 3′->CdHIS1 for/R primer contains 80 bp of homology to the upstream region of the C. dubliniensis *HIS1* cassette. The CdHIS1 for->BRG1 3′/F primer contains 80 bp of homology to the downstream region of the C. albicans *BRG1* gene. The CdHIS1 rev->UME6 3′/R primer contains 80 bp of homology to the downstream region of the *UME6* locus.

### PCR genotyping.

Genotyping of the *UME6*:*BRG1* synthetic locus was performed using four different pairs of primers. Amplification using these primer pairs yielding a DNA segment of the correct size is indicative of the expected homologous integration event. Integration of *UME6* at the *UME6* locus was confirmed using primers UME6 FarUP/F and UME6 Int/R. Integration of *BRG1* downstream of *UME6* was confirmed using primers UME6 2346 Int/F and BRG1 Int/R. Integration of the C. dubliniensis *HIS1* cassette was confirmed using primers BRG1 Int/F and HIS1 Check Int/R, as well as primers HIS1 CRIME/F and UME6 FarDOWN/R.

Homozygosity was confirmed using primers UME6 MGC/F and UME6 downstream/R, which yields either a 1.1-kbp product if a *ume6*Δ::*r1* allele is present or a 3.3-kbp product if a *UME6* allele is present.

### Single guide RNA cassette construction.

Fusion PCR was used to assemble sgRNA cassettes by previously described protocols (13, 19). The *arg4*::*dpl200* sgRNA cassette was prepared using primers sgRNA/F arg4::dpl200 and SNR52/R arg4::dpl200 and targets the sequence TCAAGAATAATCCCGCTGCT. The *ume6*::r sgRNA cassette was prepared using primers sgRNA/F ume6::r and SNR52/R ume6::r and targets the sequence TTAATCCACTGTATAACTCG.

### RNA preparation and NanoString analysis.

Overnight cultures of the relevant strains were inoculated into 25 ml of YPD to a final optical density at 600 nm (OD_600_) of 0.2 in 250-ml Erlenmeyer flasks. Cultures were then allowed to grow at 4 h with vigorous shaking (200 rpm) at 37°C, then collected by vacuum filtration, and stored at −80°C until ready for extraction. RNA was extracted from frozen cell cultures using the Qiagen RNeasy minikit (catalog no. 74104) with modifications as described previously (11).

Nanostring analysis of RNA samples was performed using previously described methods (11). The Nanostring code set contained 166 gene targets associated with environmental response pathways and 15 gene targets of interest for a variety of reasons (11).

### Biofilm and filamentation assay imaging.

Biofilms were grown according to previously described protocols with the following modifications (11). Overnight cultures grown at 30°C were inoculated into 2 ml YPD in wells containing a silicone square (Bentec Medical Inc.) (1.5 by 1.5 cm) and allowed to adhere to the silicone square for 90 min in an incubator-shaker at 37°C with shaking at 60 rpm. The squares were then washed by brief immersion into a well containing 2 ml of phosphate-buffered saline (PBS) and then placed into a fresh well containing 2 ml YPD. After 24 h, biofilms were fixed using 2 ml of 4% formaldehyde and 2.5% glutaraldehyde in 1× PBS on an orbital shaker for 1 h at 60 rpm. The biofilms were then stained for 24 h with concanavalin A–Alexa Fluor 594 conjugate (Life Technologies) diluted to 25 µg/ml in PBS, before being imaged using a slit-scan confocal optical unit on a Zeiss Axiovert 200 microscope with a 40× 0.85-numerical-aperture (NA) oil immersion objective. The imaging processes are described in detail elsewhere (30).

The filamentation assay was performed using strains inoculated into 5 ml YPD in glass test tubes and grown for 4 h at 37°C in a roller drum at maximum speed. Following growth, the cells were fixed and stained with Calcofluor white. An aliquot of cells was then imaged using conventional fluorescence microscopy on a Zeiss Axio observer Z.1 fluorescence microscope and a 20× 0.8-NA objective.

### Software.

Colony counts were collected using OpenCFU software (31). Microscopy images were compiled with ImageJ (32), Photoshop CS6, and Microsoft PowerPoint 2010. RNA counts were analyzed in Microsoft Excel 2010 and GraphPad Prism 7. Heat maps were generated using MeV software (33) and then arranged with Photoshop.

### Data availability.

Nanostring transcription profiling data may be found in Table S3.

10.1128/mSphere.00169-18.3 TABLE S3  Nanostring measurements of RNA levels. Raw data, normalized probe counts, and gene expression ratios are given on three different tabs. Download TABLE S3, XLSX file, 0.2 MB. Copyright © 2018 Huang et al. 2018 Huang et al. This content is distributed under the terms of the Creative Commons Attribution 4.0 International license.

## References

[1] Falkow S 1988 Molecular Koch’s postulates applied to microbial pathogenicity. Rev Infect Dis 10(Suppl 2):S274–S276. http://www.jstor.org/stable/4454582. 3055197 10.1093/cid/10.supplement_2.s274

[2] Baryshnikova A, Costanzo M, Myers CL, Andrews B, Boone C 2013 Genetic interaction networks: toward an understanding of heritability. Annu Rev Genomics Hum Genet 14:111–133. doi:10.1146/annurev-genom-082509-141730. 23808365

[3] Bharucha N, Chabrier-Rosello Y, Xu T, Johnson C, Sobczynski S, Song Q, Dobry CJ, Eckwahl MJ, Anderson CP, Benjamin AJ, Kumar A, Krysan DJ 2011 A large-scale complex haploinsufficiency-based genetic interaction screen in Candida albicans: analysis of the RAM network during morphogenesis. PLoS Genet 7:e1002058. doi:10.1371/journal.pgen.1002058. 22103005 PMC3084211

[4] Bockmühl DP, Krishnamurthy S, Gerads M, Sonneborn A, Ernst JF 2001 Distinct and redundant roles of the two protein kinase A isoforms Tpk1p and Tpk2p in morphogenesis and growth of Candida albicans. Mol Microbiol 42:1243–1257. doi:10.1046/j.1365-2958.2001.02688.x. 11886556

[5] Braun BR, Johnson AD 2000 TUP1, CPH1 and EFG1 make independent contributions to filamentation in Candida albicans. Genetics 155:57–67. 10790384 10.1093/genetics/155.1.57 PMC1461068

[6] Davis D, Wilson RB, Mitchell AP 2000 RIM101-dependent and -independent pathways govern pH responses in Candida albicans. Mol Cell Biol 20:971–978. doi:10.1128/MCB.20.3.971-978.2000. 10629054 PMC85214

[7] Lermann U, Morschhäuser J 2008 Secreted aspartic proteases are not required for invasion of reconstituted human epithelia by Candida albicans. Microbiology 154:3281–3295. doi:10.1099/mic.0.2008/022525-0. 18957582

[8] Li X, Robbins N, O’Meara TR, Cowen LE 2017 Extensive functional redundancy in the regulation of Candida albicans drug resistance and morphogenesis by lysine deacetylases Hos2, Hda1, Rpd3 and Rpd31. Mol Microbiol 103:635–656. doi:10.1111/mmi.13578. 27868254 PMC5296215

[9] Lo HJ, Köhler JR, DiDomenico B, Loebenberg D, Cacciapuoti A, Fink GR 1997 Nonfilamentous C. albicans mutants are avirulent. Cell 90:939–949. doi:10.1016/S0092-8674(00)80358-X. 9298905

[10] Shapiro RS, Chavez A, Porter CBM, Hamblin M, Kaas CS, DiCarlo JE, Zeng G, Xu X, Revtovich AV, Kirienko NV, Wang Y, Church GM, Collins JJ 2018 A CRISPR-Cas9-based gene drive platform for genetic interaction analysis in Candida albicans. Nat Microbiol 3:73–82. doi:10.1038/s41564-017-0043-0. 29062088 PMC5832965

[11] Woolford CA, Lagree K, Xu W, Aleynikov T, Adhikari H, Sanchez H, Cullen PJ, Lanni F, Andes DR, Mitchell AP 2016 Bypass of Candida albicans filamentation/biofilm regulators through diminished expression of protein kinase Cak1. PLoS Genet 12:e1006487. doi:10.1371/journal.pgen.1006487. 27935965 PMC5147786

[12] Vyas VK, Barrasa MI, Fink GR 2015 A Candida albicans CRISPR system permits genetic engineering of essential genes and gene families. Sci Adv 1:e1500248. doi:10.1126/sciadv.1500248. 25977940 PMC4428347

[13] Min K, Ichikawa Y, Woolford CA, Mitchell AP 2016 Candida albicans gene deletion with a transient CRISPR-Cas9 system. mSphere 1:e00130-16. doi:10.1128/mSphere.00130-16. 27340698 PMC4911798

[14] Wilson RB, Davis D, Mitchell AP 1999 Rapid hypothesis testing with Candida albicans through gene disruption with short homology regions. J Bacteriol 181:1868–1874. 10074081 10.1128/jb.181.6.1868-1874.1999 PMC93587

[15] Banerjee M, Thompson DS, Lazzell A, Carlisle PL, Pierce C, Monteagudo C, López-Ribot JL, Kadosh D 2008 UME6, a novel filament-specific regulator of Candida albicans hyphal extension and virulence. Mol Biol Cell 19:1354–1365. doi:10.1091/mbc.E07-11-1110. 18216277 PMC2291399

[16] Cleary IA, Lazzell AL, Monteagudo C, Thomas DP, Saville SP 2012 BRG1 and NRG1 form a novel feedback circuit regulating Candida albicans hypha formation and virulence. Mol Microbiol 85:557–573. doi:10.1111/j.1365-2958.2012.08127.x. 22757963 PMC3402693

[17] Du H, Guan G, Xie J, Sun Y, Tong Y, Zhang L, Huang G 2012 Roles of Candida albicans Gat2, a GATA-type zinc finger transcription factor, in biofilm formation, filamentous growth and virulence. PLoS One 7:e29707. doi:10.1371/journal.pone.0029707. 22276126 PMC3261855

[18] Nobile CJ, Fox EP, Nett JE, Sorrells TR, Mitrovich QM, Hernday AD, Tuch BB, Andes DR, Johnson AD 2012 A recently evolved transcriptional network controls biofilm development in Candida albicans. Cell 148:126–138. doi:10.1016/j.cell.2011.10.048. 22265407 PMC3266547

[19] Huang MY, Mitchell AP 2017 Marker recycling in Candida albicans through CRISPR-Cas9-induced marker excision. mSphere 2:e00050-17. doi:10.1128/mSphere.00050-17. 28317025 PMC5352831

[20] Ma H, Kunes S, Schatz PJ, Botstein D 1987 Plasmid construction by homologous recombination in yeast. Gene 58:201–216. doi:10.1016/0378-1119(87)90376-3. 2828185

[21] Colot HV, Park G, Turner GE, Ringelberg C, Crew CM, Litvinkova L, Weiss RL, Borkovich KA, Dunlap JC 2006 A high-throughput gene knockout procedure for Neurospora reveals functions for multiple transcription factors. Proc Natl Acad Sci U S A 103:10352–10357. doi:10.1073/pnas.0601456103. 16801547 PMC1482798

[22] Gibson DG, Benders GA, Andrews-Pfannkoch C, Denisova EA, Baden-Tillson H, Zaveri J, Stockwell TB, Brownley A, Thomas DW, Algire MA, Merryman C, Young L, Noskov VN, Glass JI, Venter JC, Hutchison CA III, Smith HO 2008 Complete chemical synthesis, assembly, and cloning of a Mycoplasma genitalium genome. Science 319:1215–1220. doi:10.1126/science.1151721. 18218864

[23] Nguyen N, Quail MMF, Hernday AD 2017 An efficient, rapid, and recyclable system for CRISPR-mediated genome editing in Candida albicans. mSphere 2:e00149-17. doi:10.1128/mSphereDirect.00149-17. 28497115 PMC5422035

[24] Childers DS, Kadosh D 2015 Filament condition-specific response elements control the expression of NRG1 and UME6, key transcriptional regulators of morphology and virulence in Candida albicans. PLoS One 10:e0122775. doi:10.1371/journal.pone.0122775. 25811669 PMC4374957

[25] Childers DS, Mundodi V, Banerjee M, Kadosh D 2014 A 5′ UTR-mediated translational efficiency mechanism inhibits the Candida albicans morphological transition. Mol Microbiol 92:570–585. doi:10.1111/mmi.12576. 24601998 PMC4032089

[26] Argimón S, Wishart JA, Leng R, Macaskill S, Mavor A, Alexandris T, Nicholls S, Knight AW, Enjalbert B, Walmsley R, Odds FC, Gow NA, Brown AJ 2007 Developmental regulation of an adhesin gene during cellular morphogenesis in the fungal pathogen Candida albicans. Eukaryot Cell 6:682–692. doi:10.1128/EC.00340-06. 17277173 PMC1865654

[27] Kim S, Nguyen QB, Wolyniak MJ, Frechette G, Lehman CR, Fox BK, Sundstrom P 2018 Release of transcriptional repression through the HCR promoter region confers uniform expression of HWP1 on surfaces of Candida albicans germ tubes. PLoS One 13:e0192260. doi:10.1371/journal.pone.0192260. 29438403 PMC5810986

[28] Hoffman CS, Winston F 1987 A ten-minute DNA preparation from yeast efficiently releases autonomous plasmids for transformation of Escherichia coli. Gene 57:267–272. doi:10.1016/0378-1119(87)90131-4. 3319781

[29] Noble SM, Johnson AD 2005 Strains and strategies for large-scale gene deletion studies of the diploid human fungal pathogen Candida albicans. Eukaryot Cell 4:298–309. doi:10.1128/EC.4.2.298-309.2005. 15701792 PMC549318

[30] Lagree K, Desai JV, Finkel JS, Lanni F 2018 Microscopy of fungal biofilms. Curr Opin Microbiol 43:100–107. doi:10.1016/j.mib.2017.12.008. 29414442

[31] Geissmann Q 2013 OpenCFU, a new free and open-source software to count cell colonies and other circular objects. PLoS One 8:e54072. doi:10.1371/journal.pone.0054072. 23457446 PMC3574151

[32] Schindelin J, Arganda-Carreras I, Frise E, Kaynig V, Longair M, Pietzsch T, Preibisch S, Rueden C, Saalfeld S, Schmid B, Tinevez JY, White DJ, Hartenstein V, Eliceiri K, Tomancak P, Cardona A 2012 Fiji: an open-source platform for biological-image analysis. Nat Methods 9:676–682. doi:10.1038/nmeth.2019. 22743772 PMC3855844

[33] Saeed AI, Sharov V, White J, Li J, Liang W, Bhagabati N, Braisted J, Klapa M, Currier T, Thiagarajan M, Sturn A, Snuffin M, Rezantsev A, Popov D, Ryltsov A, Kostukovich E, Borisovsky I, Liu Z, Vinsavich A, Trush V, Quackenbush J 2003 TM4: a free, open-source system for microarray data management and analysis. Biotechniques 34:374–378. 12613259 10.2144/03342mt01

